# Tuberculin Skin Tests versus Interferon-Gamma Release Assays in Tuberculosis Screening among Immigrant Visa Applicants

**DOI:** 10.1155/2014/217969

**Published:** 2014-03-06

**Authors:** Stella O. Chuke, Nguyen Thi Ngoc Yen, Kayla F. Laserson, Nguyen Huu Phuoc, Nguyen An Trinh, Duong Thi Cam Nhung, Vo Thi Chi Mai, An Dang Qui, Hoang Hoa Hai, Le Thien Huong Loan, Warren G. Jones, William C. Whitworth, J. Jina Shah, John A. Painter, Gerald H. Mazurek, Susan A. Maloney

**Affiliations:** ^1^Northrop Grumman Information Systems Sector, 2800 Century Parkway NE, Atlanta, GA 30345, USA; ^2^Division of Tuberculosis Elimination, Centers for Disease Control and Prevention (CDC), Mail Stop E-10, 1600 Clifton Road NE, Atlanta, GA 30333, USA; ^3^Cho Ray Hospital, 201B Nguyen Chi Thanh Street, District 5, Ho Chi Minh City, Vietnam; ^4^Center for Global Health, CDC, Mail Stop D-68, 1600 Clifton Road NE, Atlanta, GA 30333, USA; ^5^International Organization for Migration (IOM), 1B Pham Ngoc Thach District 1, Ho Chi Minh City, Vietnam; ^6^International Organization for Migration (IOM), P.O. Box 55040, Westlands, Nairobi 00200, Kenya; ^7^Division of Global Migration and Quarantine, CDC, Mail Stop E-03, 1600 Clifton Road NE, Atlanta, GA 30333, USA; ^8^Department of Family & Community Medicine, University of California, San Francisco, 500 Parnassus Avenue, MU 3E, San Francisco, CA 94143, USA; ^9^Genentech, Inc., 1 DNA Way, South San Francisco, CA 94080, USA

## Abstract

*Objective*. Use of tuberculin skin tests (TSTs) and interferon gamma release assays (IGRAs) as part of tuberculosis (TB) screening among immigrants from high TB-burden countries has not been fully evaluated. *Methods*. Prevalence of *Mycobacterium tuberculosis* infection (MTBI) based on TST, or the QuantiFERON-TB Gold test (QFT-G), was determined among immigrant applicants in Vietnam bound for the United States (US); factors associated with test results and discordance were assessed; predictive values of TST and QFT-G for identifying chest radiographs (CXRs) consistent with TB were calculated. *Results*. Of 1,246 immigrant visa applicants studied, 57.9% were TST positive, 28.3% were QFT-G positive, and test agreement was 59.4%. Increasing age was associated with positive TST results, positive QFT-G results, TST-positive but QFT-G-negative discordance, and abnormal CXRs consistent with TB. Positive predictive values of TST and QFT-G for an abnormal CXR were 25.9% and 25.6%, respectively. *Conclusion*. The estimated prevalence of MTBI among US-bound visa applicants in Vietnam based on TST was twice that based on QFT-G, and 14 times higher than a TST-based estimate of MTBI prevalence reported for the general US population in 2000. QFT-G was not better than TST at predicting abnormal CXRs consistent with TB.

## 1. Introduction

Tuberculosis (TB) is the single leading cause of death from a curable infectious disease [1], with a global death toll of 1.4 million persons in 2011 [2]. The global incidence of TB has increased from approximately 1.2 million in 1995 [3] to an estimate of 8.7 million in 2011 [2]. About one-third of the world's population is infected with the causative organism,* Mycobacterium tuberculosis* (MTB) [4]. Without intervention, 5 to 10% of those latently infected with MTB are expected to develop active and infectious TB during their lifetime [5].

Global migration has had an increasingly important effect on the epidemiology of TB in the United States and other countries. Although the overall number of new TB cases is decreasing in the US, there has been a significant increase in the proportion of US TB cases in persons born outside the US, with foreign-born persons accounting for 57% of new TB cases in 2006 [6] and 62% in 2011 [7]. To decrease the risk of TB transmission and to improve TB diagnosis and treatment outcomes, immigrant visa applicants are screened for infectious TB [8–11]. For visa applicants ≥15 years of age residing outside the US, medical evaluation includes screening with a chest radiograph (CXR), followed by sputum examination if the CXR is suggestive of TB [12]. For visa applicants already residing within the US and for those outside the US who are <15 years of age, medical evaluation includes screening with either a tuberculin skin test (TST) or an interferon gamma release assay (IGRA), followed by a CXR if either test is positive [12]. However, the utility of TSTs and IGRAs among immigrants and refugees from high TB-burden countries, such as Vietnam, has not been fully evaluated.

Employing recently developed IGRAs may facilitate TB screening. IGRAs such as the QuantiFERON-TB test (QFT) and the QuantiFERON-TB Gold test (QFT-G) were developed as aids for diagnosing MTB infection (MTBI) which includes both latent TB infection (LTBI) and infection manifesting as active TB disease [13]. IGRAs may be completed with a single patient visit, may be less subjective, and may be performed more rapidly than TST [13]. QFT uses tuberculin purified protein derivative (PPD) as the TB antigen and includes an* M. avium *PPD as a control for reactivity to nontuberculous mycobacteria (NTM) [14]. QFT-G assesses reactivity to two MTB proteins, early secretory antigenic target 6 (ESAT-6) and culture filtrate protein 10 (CFP-10) [13]. ESAT-6 and CFP-10 are absent from all bacille Calmette-Guerin (BCG) vaccine strains and most NTM [15]. QFT-G may be more specific than TST and QFT because of less cross-reactivity with BCG and NTM [16]. The QuantiFERON-TB Gold In-Tube test (QFT-GIT) was introduced after this study was initiated and QFT-GIT was conceptualized in part as a result of this study. QFT-GIT facilitates testing by including antigens or control reagents in special tubes used to collect blood for the test [13].

In 2011, immigrants from Vietnam accounted for 5% of US TB cases and 8% of TB cases among foreign-born US residents [7]. Despite meeting WHO targets of 70% smear-positive case detection and 85% cure rates in 1996, Vietnam remains a high TB burden country with an estimated prevalence of 323 cases per 100, 000 persons in 2011 [2]. This high burden of disease increases the risk of TB transmission in Vietnam [17, 18] and in other developed nations to which immigrants may be resettling [19]. Vietnam has maintained its policy of BCG vaccination of children at birth since 1924 [20–22] and coverage is high (93.7% by a 2007 estimate) [22].

The objectives of this study conducted among immigrant visa applicants in Vietnam were to (1) determine the prevalence of MTBI based on TST and QFT-G; (2) assess the association of various factors (including* M. avium* reactivity as measured by QFT) with TST and QFT-G results and discordance; and (3) compare the predictive values of TST and QFT-G at identifying CXRs consistent with TB.

## 2. Methods

This study was part of a larger study examining the efficacy of TB screening among immigrant visa applicants [23, 24] and included a systematic sample of adult immigrant visa applicants. Subjects were recruited on Wednesdays from among adults (age ≥ 18 years) presenting for immigrant medical examinations (MEs) at Cho Ray Hospital in Ho Chi Minh City, Vietnam, from June 12, 2002, to March 12, 2003. MEs are performed in clinics at this hospital five days a week. Wednesday was chosen as the day to recruit because of convenience. MEs are performed according to the 1991 technical instructions published by CDC [10]. All subjects provided written consent. The study was approved by human subject protection committees at CDC, Cho Ray Hospital, Pasteur Institute, and Pham Ngoc Thach National Tuberculosis and Lung Disease Center.

Information relating to nativity, gender, medical history, findings on physical examination, HIV test results, and CXR findings was abstracted from standardized ME forms. Subjects were asked for additional information related to prior TB disease or treatment, TB exposure, TB symptoms, and BCG vaccination. Each subject was examined for the presence of a BCG scar. Subjects with CXR findings consistent with TB were asked to provide sputum on 3 consecutive days. Uncentrifuged sputa were examined for AFB using both Auramine O fluorescence and Ziehl-Neelsen staining methods [25]. Sputa from subjects were decontaminated and digested with oxalic acid (due to pseudomonas in the town water supply) and cultured for mycobacteria using the BACTEC 460 system (Becton, Dickinson and Company, Franklin Lakes, NJ) and Lowenstein-Jensen slants as previously described [24].

Blood samples for QFT and QFT-G were obtained before PPD injections. TSTs were administered by the Mantoux method using 0.1 mL (5 TU) of Tubersol PPD (Connaught Laboratories Inc., Toronto, ON). TST induration was measured 48 to 72 hours after PPD injection by trained health care workers who were blinded to QFT and QFT-G results. Indurations ≥10 mm were interpreted as positive. QFT and QFT-G were performed and interpreted as previously described [26] by staff blinded to results of other tests. TB response by QFT-G was the larger of the interferon gamma responses to CFP-10 or ESAT-6. CXRs were interpreted by panel physicians who were blinded to TST, QFT, and QFT-G results but were aware of other clinical findings.

### 2.1. Statistical Methods

Demographic, clinical, and laboratory data from each subject were entered into a Microsoft Access Database (Microsoft Corp., Redmond, WA). All data analyses were performed using SPSS (version 15.0; SPSS Inc., Chicago, IL).* M. avium* reactivity was coded as “positive” when QFT results were “Negative for* M. tuberculosis* infection with* M. avium* reactivity” as defined previously [26]; otherwise it was coded as “negative.” CXRs were coded as “positive” if findings were consistent with TB according to published criteria [27]. CXRs were coded as “negative” if they were normal or showed only abnormalities that were not consistent with TB (e.g., fractured rib or cardiac enlargement).

Prevalence of MTBI was calculated among those who had TST, QFT, QFT-G, and CXRs completed. Test agreement, positive predictive value (PPV), and negative predictive value (NPV) were calculated among subjects with determinate QFT-G results who had TST and CXR completed. Agreement beyond chance was assessed using Cohen's Kappa coefficient (*κ*) with a *κ* > 0.75 representing excellent agreement, 0.40–0.75 representing fair to good agreement, and <0.40 representing poor agreement [28]. PPVs or NPVs were compared using a predictive value statistic that utilized the Wald procedure [29]. The McNemar test was used to compare estimates of prevalence [30]. Tests for significance were 2-sided and considered statistically significant at a *P* value of < 0.05. Discordance between TST and QFT-G was classified as “TST positive but QFT-G negative,” or “TST negative but QFT-G positive.” Discordance between CXR and TST and between CXR and QFT-G was classified in a similar manner.

Univariate and multivariate logistic regressions were used to assess association of the subject characteristics listed in Table 1 with test results and with test discordance. Multivariate models were created using factors with *P* values < 0.2 in univariate analysis and <0.05 in stepwise logistic regression until the best fitting, parsimonious model was identified. Model fit was evaluated using the Hosmer-Lemeshow test [31]. No interactions between subject characteristics were considered to be of interest* a priori*.

## 3. Results

**Figure 1:**
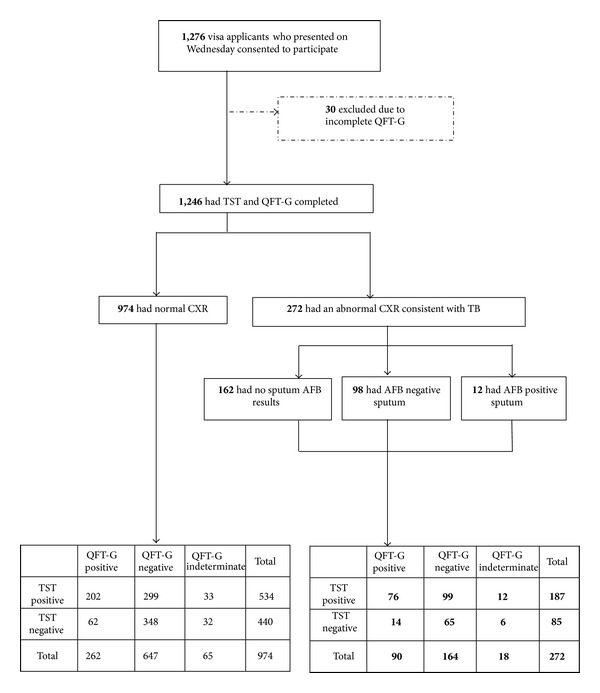
Participation diagram. Immigrant visa applicants who presented to Cho Ray Hospital on Wednesdays were asked to participate. QFT-G: QuantiFERON-TB Gold test; TST: tuberculin skin test; CXR: chest radiograph; TB: tuberculosis; AFB: acid fast bacillus.

As depicted in Figure 1, of the 1,276 subjects in the systematic sample who consented, 30 (2.4%) were excluded because QFT-G was not completed. Characteristics of those excluded (data not shown) did not differ statistically from characteristics of the remaining 1,246 subjects who had QFT-G, TST, and CXR completed (Table 1). As summarized in Table 1, CXR findings for 272 (21.8%) subjects were consistent with TB; 362 (29.1%) subjects had* M. avium* reactivity by QFT; 721 (57.9%) subjects had positive TST results with induration ≥ 10 mm; 352 (28.3%) subjects had positive QFT-G results and 83 (6.7%) subjects had indeterminate QFT-G results. Positive TST results were more prevalent than positive QFT-G results, and both were more prevalent than positive CXRs (all *P* values ≤ 0.001). Of the 272 subjects with CXRs consistent with TB, 110 (40%) provided sputum and 12 had AFB seen on smear. Culture results were available for 67 subjects and 16 had positive cultures for* M. tuberculosis*. None of the 16 subjects with positive cultures for* M. tuberculosis* had a prior history of TB; all had a positive TST; and 7 (43.8%) had a positive QFT-G.

Mean, median, and interquartile range for TB response values stratified by QFT-G interpretation are shown in Table 2. TB responses were within 0.25 IU/mL of the 0.35 IU/mL cutoff for 70 (19.9%) of 352 QFT-G interpreted as positive and 192 (23.7%) of 811 QFT-G interpreted as negative.

As shown in Table 3, positive CXR results were associated with increased age, male sex, and abnormal chest examination; positive TST results (i.e., induration ≥ 10 mm) were associated with increased age, male sex, and prior BCG vaccination (assessed by self-report or by scar); and positive QFT-G results were associated with increased age.* M. avium* reactivity was inversely associated with positive TST results and positive QFT-G results. Seven subjects had prior TB, all of whom had CXRs consistent with TB, 6 (85.7%) of whom had a positive TST, and 2 (28.6%) had a positive QFT-G. “Prior TB” was not included as a variable in our final multivariate models because all seven subjects with prior TB had CXR findings consistent with TB disease and this prevented convergence of the model, or “Prior TB” was not associated with TST or QFT-G results in univariate analyses (*P* values > 0.2).

Indeterminate QFT-G results were not associated with any subject characteristics examined but were inversely associated with* M. avium* reactivity with an odds ratio of 0.35 (95% CI: 0.19–0.68). Further analysis showed that TST induration ≥ 15 mm was not associated with BCG vaccination (*P* = 0.46), but associations with age (*P* < 0.01) and male sex (*P* = 0.03) remained significant in our multivariate model and induration ≥ 15 mm also remained inversely associated with* M. avium* reactivity (*P* < 0.01).

When limited to subjects with determinate QFT-G results (all of whom had TST and CXR completed), overall agreement between TST and QFT-G, between CXR and TST, and between CXR and QFT-G was 59.4%, 50.1%, and 63.4%, respectively, (Table 4) and agreement beyond chance was poor. The PPVs of TST and QFT-G for a positive CXR were 25.9% (95% CI: 22.6%–29.2%) and 25.6% (95% CI: 21.0%–30.1%), respectively. The NPVs of TST and QFT-G for a negative CXR were 83.8% (95% CI: 80.5%–87.1%) and 79.8% (95% CI: 77.0%–85.6%), respectively. While PPVs for TST and QFT-G were similar (*P* = 0.87), the NPV for TST was greater than the NPV for QFT-G (*P* < 0.01).

As shown in Table 5, there were 398 (34.2%) subjects with positive TST but negative QFT-G results, and this discordance was associated with increased age, male sex, and prior BCG vaccination. There were 74 (6.4%) subjects with negative TST but positive QFT-G results and none of the subject characteristics examined were associated with this discordance.

As shown in Table 6, there were 79 (6.8%) subjects with positive CXR but negative TST results, and this discordance was associated with increased age and other major medical conditions. There were 501 (43.1%) subjects with negative CXR but positive TST results. This discordance was associated with increased age, male sex, and BCG vaccination and inversely associated with* M. avium* reactivity.

As shown in Table 7, there were 164 (14.1%) subjects with positive CXR but negative QFT-G results, and this discordance was associated with increased age, history of TB disease, and abnormal chest examination. There were 262 (22.5%) subjects with negative CXR but positive QFT-G results and this discordance was inversely associated with age.

## 4. Discussion

Our study demonstrates high MTBI prevalence among US-bound immigrant applicants as compared to the general US population. Of the 1,246 immigrant applicants included in our systematic sample from June 12, 2002, to March 12, 2003, 58% had a positive TST and 28% had a positive QFT-G. Both of these measures of MTBI prevalence are higher than the 4.2% prevalence reported among the general US population in 2000 based on TST [32]. The prevalence of MTBI among visa immigrant applicants based on the TST is 14 times higher than the prevalence in the general US population using the same test. The prevalence among immigrant applicants based on QFT-G is 7 times higher than the TST-based estimate in the general US population. Estimates among other segments of the Vietnamese population also suggest high rates of MTBI. TST was positive in 61.1% of healthcare workers in Hanoi, Vietnam [33]. In 2005-2006, 6 to 7% of school children 6 to 9 years of age in Ho Chi Minh Province had TST reactions ≥ 15 mm [34]. As there is no diagnostic gold standard for documenting most MTBIs (including LTBI, culture-negative pulmonary TB, and most extrapulmonary TB), the true prevalence of MTBI is unknown.

TB screening of immigrant visa applicants overseas relies heavily on CXRs [10, 11]. All adult immigrant applicants residing outside the US are required to have a CXR and are not required to have a test for MTBI. In contrast, immigrant applicants already residing in the US are screened for MTBI with a TST or IGRA, and those with evidence of MTBI are required to have a CXR. Those with CXR findings compatible with TB are evaluated further for infectious TB by sputum AFB smear and/or culture. Screening by CXR is intended to identify applicants with infectious TB and has been effective [35]. Our study demonstrated that substantially fewer adult immigrant applicants had evidence of TB on CXR (22%) than had a positive TST (58%) or a positive QFT-G (28%). Thus, the current screening algorithm may leave a substantial number of immigrant applicants with LTBI undetected, untreated, and at increased risk of subsequently developing TB. In addition, people with extrapulmonary TB may have a normal CXR, [11, 24] and these persons may be missed using the current screening algorithm that relies on CXR.

Characteristics associated with a positive TST, QFT-G, or CXR (i.e., an abnormal CXR consistent with TB) were different (Table 3). Of the multiple subject characteristics associated with positive test results, only one (increased age) was associated with positive results by all three tests. The association with age suggests accumulating MTBI. Alternatively, older persons may have lived when TB was more prevalent and the association with age may represent a cohort effect. For CXR, this may also reflect cumulative scarring, disease, or infection due to other organisms or illness, but the associations with age persisted after adjustment for other factors.

Agreement between TST and QFT-G, CXR and TST, and CXR and QFT-G was poor (Table 4). Various subject characteristics are associated with the different types of discordance (Tables 5, 6, and 7). Some discordance may reflect the accumulation or presence of CXR lesions due to illnesses other than TB, as suggested by associations of discordance with age, other major medical conditions, and abnormal chest exams (Tables 6 and 7). Poor agreement between TST and QFT-G has been described in other studies [16, 36–39]. TST positive but QFT-G negative discordance has been attributed to false-positive TST results following BCG vaccination [36, 40] or NTM exposure [38] and false-negative IGRA results [16, 37, 41–43]. TST negative but QFT-G positive discordance has been associated with immune suppression with lower TST sensitivity [26, 44], false-positive QFT-G results as described among low risk healthcare workers [45], or unexplained [42] as seen with the present study (Table 5).

BCG may contribute to false-positive TST results because it produces many of the antigens produced by MTB and that are present in the tuberculin PPD. However, BCG does not produce the antigens used in QFT-G (i.e., ESAT-6 and CFP-10) [15]. BCG is an attenuated* M. bovis* strain so that vaccination and infection do not typically cause disease or CXR lesions. In our study, BCG vaccination was associated with (1) positive TST results, (2) TST positive but QFT-G negative discordance, and (3) CXR negative but TST positive discordance (using a 10 mm cutoff). These findings support the hypothesis that some TST reactions ≥ 10 mm may be due to BCG vaccination. They appear to disagree with conclusions of others that induration ≥ 10 mm is unlikely due to BCG administered at infancy and more than 10 years prior to the TST [46] because BCG is given only at birth in Vietnam [21] and the youngest subject enrolled in our study was 18 years of age. Further analysis of our data showed that TST induration ≥ 15 mm was not associated with BCG vaccination. This suggests that, in our study population, TST induration < 15 mm (including those with induration of 10 to 15 mm) may be due to BCG vaccination, but induration ≥ 15 is more likely due to MTBI than to BCG. Prior BCG vaccination does not account for all TST positive but QFT-G negative discordance since almost half (46%) of the TST results ≥ 15 mm were in subjects who were not vaccinated. Additionally, this type of discordance was associated with age and it is unlikely that the degree of discordance due to BCG would increase with age.

We hypothesized that NTM infection might cause false-positive TST results and contribute to TST positive but QFT-G negative discordance. However,* M. avium* reactivity was not associated with TST positive but QFT-G negative discordance. Additionally, positive TST results and positive QFT-G results were less common among participants with* M. avium* reactivity as measured by QFT (Table 3). One possibility is that* M. avium* responsiveness may offer some protection against MTB infection. This is in contrast to findings in the US where* M. avium* reactivity was associated with TST induration ≥ 10 mm and with TST positive but QFT-G negative discordance, but not with positive QFT-G results [16]. These findings were attributed to cross-reactivity with* M. avium* causing false-positive TST reactions.

We observed an association between male sex and (1) positive TST results, (2) CXR findings consistent with TB, (3) TST positive but QFT-G negative discordance, and (4) negative CXR but positive TST results. Male predominance among those infected with MTB and those with TB disease has been described previously and may be due to gender differences in occupational or social MTB exposure [17, 47, 48]. Smoking may increase TB risk and smoking among Vietnamese males is common but among females is rare [49, 50]. These findings and the observation that almost half (46%) of the TST results ≥ 15 mm were QFT-G negative suggest that some of the discordance is due to false-negative QFT-G results as suggested previously [16, 37, 41–43].

Neither TST nor QFT-G performed well as predictors of an abnormal CXR consistent with TB in this population. The PPVs of both TST and QFT-G for an abnormal CXR were extremely low (25.9% and 25.6%, resp.), which may not be surprising because we expect that a large number of those with a normal CXR may have LTBI that has not progressed to TB disease. The NPVs of TST and QFT-G were also less than optimal (83.8% and 79.8%, resp.), possibly because many CXR abnormalities are not due to TB. Of more importance is the accuracy of these tests for active TB disease. Too few culture results were available to reliably assess the sensitivity of TST or QFT-G for culture-confirmed TB in our systematic sample. A review of studies comparing TST and QFT-G among people with active TB reports a pooled sensitivity of 77% and 78%, respectively [51].

We recognize several limitations with this study. First, while immigrant applicants from all regions of Vietnam are evaluated at the Cho Ray Hospital Clinic, it is unknown if the prevalence of MTBI in the general Vietnamese population is similar to the prevalence among immigrant applicants. Second, selection bias could have occurred due to our restriction of enrollment to applicants presenting on Wednesday. However, none of our experiences or data suggested an enrollment bias. Third, recall bias may limit studies that rely on questionnaires, especially when asking about BCG vaccination. In Vietnam, with universal BCG vaccination and estimated vaccination coverage of 93.7%, we might have expected higher reported BCG vaccination rates than the 41% that we found among the study participants. On the other hand, our findings might be related to the specific visa applicant pool we evaluated (in terms of such variables as birth place/province within Vietnam, mobility, age, or socioeconomic strata); our study collected information on the presence of BCG scars as well, and we found that for every individual with a positive BCG vaccination history, there was a corresponding scar seen on physical examination. If we assumed that BCG vaccination was universal (but masked by recall bias linked to absence of a visible scar) and removed BCG from our models, the magnitude of the adjusted odds ratios for the remaining variables (e.g., age, sex, and* M. avium* reactivity) changed little and there was no addition or loss of variables with significance (data not shown). QFT and QFT-G have been supplanted by the newer QuantiFERON-TB Gold In-Tube test (QFT-GIT). Similar study outcomes would be expected with QFT-GIT as compared to QFT-G because of similar sensitivity and specificity [51]. Availability of QFT for this study allowed assessment of* M. avium* reactivity which could no longer be done with a commercial assay.

In conclusion, the estimated prevalence of MTBI among US-bound visa applicants in Vietnam based on TST was twice that based on QFT-G and 14 times higher than a TST-based estimate of MTBI prevalence reported for the general US population at approximately the same time. QFT-G was not better than TST at predicting abnormal CXRs consistent with TB.

## Figures and Tables

**Table 1:** Subject characteristics and test results of US-bound immigrant visa applicants in Vietnam.

Characteristics	
Subjects evaluated:	*N* = 1246
Age in years:	
Mean/median	38.5/37.5
Minimum/maximum	18.0/86.0
Sex: number (%)	
Female	842 (67.6)
Male	404 (32.4)
Country of birth: *n* (%)	
Vietnam	1235 (99.1)
Other (China or Cambodia)	11 (0.9)
Prior history of TB	7 (0.6)
Reported prior contact with a TB patient: *n* (%)	
Any contact	96 (7.7)
Household contact	93 (7.5)
TB symptoms*: *n* (%)	3 (0.2)
Other major medical conditions**: *n* (%)	17 (1.4)
BCG vaccination status***: *n* (%)	
Not vaccinated	735 (59.0)
Vaccinated	511 (41.0)
HIV test results: *n* (%)	
Negative	1239 (99.4)
Positive	7 (0.6)
Chest examination: *n* (%)	
Normal	1239 (99.4)
Abnormal	7 (0.6)
Chest radiograph: *n* (%)	
Normal or not consistent with TB****	974 (78.2)
Findings consistent with TB	272 (21.8)
TST results: *n* (%)	
<5 mm	278 (22.3)
5 to 9 mm	247 (19.8)
10 to 14 mm	403 (32.3)
≥15 mm	318 (25.5)
QFT results: *n* (%)	
Negative for MTB infection without *M. avium* reactivity	234 (18.8)
Negative for MTB infection with *M. avium* reactivity	362 (29.1)
Positive for MTB infection	564 (45.3)
Indeterminate	86 (6.9)
QFT-G results: *n* (%)	
Negative	811 (65.1)
Positive	352 (28.3)
Indeterminate	83 (6.7)

*n*: number in subset of *N*; TB: tuberculosis; BCG: bacille Calmitte-Guérin; HIV: human immunodeficiency virus; MTB: *Mycobacteria tuberculosis*; TST: tuberculin skin test; QFT: QuantiFERON-TB test; QFT-G: QuantiFERON-TB Gold test.

*TB Symptoms included cough, dyspnea, fever, unintended weight loss, and hemoptysis.

**Other major medical conditions included diabetes, renal failure, silicosis, gastrectomy, and malignancies.

***Reported BCG status based on interview. All subjects reporting BCG vaccination also had scars compatible with vaccination history.

****Chest radiographs were normal for 967 subjects while 7 subjects had abnormal chest radiographs with lesions not consistent with TB (e.g., fractured rib or cardiac enlargement).

**Table 2:** Mean, median, and interquartile range for TB response.

	Negative QFT-G	Positive QFT-G
Count	811	352
Mean (IU/mL)	0.05	3.91
Median (IU/mL)	0.30	1.53
Interquartile range (IU/mL)	0.10	3.60

QFT-G: QuantiFERON-TB Gold test.

**Table 3:** Subject characteristics associated with chest radiographs consistent with tuberculosis; TST induration ≥ 10 mm; or positive QFT-G results.

Characteristic	*N*	CXR consistent with tuberculosis		TST induration ≥ 10 mm		Positive QFT-G	
		*n*	aOR* (95% CI)	*n*	aOR* (95% CI)	*n*	aOR* (95% CI)
Total	1,246	272		721		352	
Age group							
18–20 years	139	9 (6.5%)	1.0	49 (35.3%)	1.0	25 (18.0%)	1.0
21–30 years	293	19 (6.5%)	1.01 (0.44–2.32)	143 (48.8%)	**2.17 (1.40–3.35)**	77 (26.3%)	**1.66 (1.002–2.759)**
31–40 years	294	36 (12.2%)	2.06 (0.96–4.42)	199 (67.7%)	**4.14 (2.67–6.43)**	89 (30.3%)	**1.97 (1.19–3.25)**
41–50 years	268	80 (29.9%)	**5.87 (2.84–12.15)**	171 (63.8%)	**3.49 (2.24–5.46)**	91 (34.0%)	**2.29 (1.39–3.79)**
51–64 years	207	99 (47.8%)	**12.94 (6.23–26.89)**	137 (66.2%)	**4.22 (2.61–6.81)**	60 (29.0%)	**1.81 (1.07–3.07)**
≥65 years	45	24 (64.4%)	**25.12 (10.06–62.70)**	22 (48.9%)	1.92 (0.95–3.92)	10 (22.2%)	1.26 (0.55–2.89)
Sex							
Female	842	148 (17.6%)	1.0	439 (52.1%)	1.0	226 (26.8%)	
Male	404	124 (30.7%)	**1.69 (1.24–2.29)**	282 (69.8%)	**2.09 (1.60–2.73)**	126 (31.2%)	N.S. and NIM
BCG status							
Not vaccinated	735	191 (26.0%)		398 (54.1%)	1.0	215 (29.3%)	
Vaccinated	511	81 (58.8%)	N.S. and NIM	323 (63.2%)	**1.62 (1.25–2.09)**	137 (26.8%)	
Chest exam							
Normal	1239	266 (21.5%)	1.0	718 (57.9%)		351 (28.3%)	
Abnormal	7	6 (85.7%)	**31.23 (3.04–320.68)**	3 (42.9%)	N.S. and NIM	1 (14.3%)	N.S. and NIM
*M. avium* reactivity							
No	884	217 (24.5%)		550 (62.2%)	1.0	268 (30.3%)	1.0
Yes	362	55 (15.2%)	N.S. and NIM	171 (47.2%)	**0.59 (0.46–0.77)**	84 (23.2%)	**0.71 (0.53–0.95)**

CXR: chest radiographs; TST: tuberculin skin test; QFT-G: QuantiFERON-TB Gold test; *n*: number in subset of *N*; aOR: adjusted odd ratio; 95% CI: 95% confidence interval; N.S.: not significant; NIM: not in model; BCG: bacille Calette-Guérin; TB: tuberculosis; QFT: QuantiFERON-TB test.

*Multivariate models were created using factors with *P* values ≤0.2 in univariate analysis and <0.05 in stepwise logistic regression until the best fitting, parsimonious model was identified. Model fit was evaluated using the Hosmer-Lemeshow test. Negative and indeterminate QFT-G results were coded as “not positive.” The variable “Prior TB” was not included in the model because all 7 subjects with prior TB had CXR findings consistent with TB disease and its inclusion prevented convergence of the model. **Bold font** indicates statistically significant adjusted odds ratios (aORs).

**Table 4:** Agreement* of TST versus QFT-G, CXR versus TST, and CXR versus QFT-G.

	TST versus QFT-G	CXR versus TST	CXR versus QFT-G
Positive/positive results; *n* (%)	278 (23.9)	175 (15.0)	90 (7.7)
Negative/negative results; *n* (%)	413 (35.5)	408 (35.1)	647 (55.6)
Positive/negative results; *n* (%)	398 (34.2)	79 (6.8)	164 (14.1)
Negative/positive results; *n* (%)	74 (6.4)	501 (43.1)	262 (22.5)
Agreement; % (95% CI)	59.4 (56.6–62.2)	50.1 (47.2–52.9)	63.4 (60.6–66.2)
Kappa coefficient; *κ* (95% CI)	0.24 (0.19–0.28)	0.09 (0.05–0.13)	0.058 (0.001–0.115)

CXR: chest radiographs; TST: tuberculin skin test; QFT-G: QuantiFERON-TB Gold test; *n*: number in subset of *N*; 95% CI: 95% confidence interval.

*Agreement was assessed among 1,163 subjects; data from 83 subjects with indeterminate QFT-G results were excluded from analysis.

**Table 5:** Subject characteristics associated with TST and QFT-G discordance.

Characteristic	*N*	TST positive but QFT-G negative		TST negative but QFT-G positive	
		*n*	aOR* (95% CI)	*n*	aOR* (95% CI)
Total	1,163**	398		74	
Age group in years					
18–20 yrs	130	27 (20.8%)	1.0	7 (5.4%)	N.S. and NIM
21–30 yrs	270	80 (29.6%)	**1.86 (1.12–3.09)**	25 (9.3%)	
31–40 yrs	279	116 (41.6%)	**2.81 (1.72–4.61)**	16 (5.7%)	
41–50 yrs	252	85 (33.7%)	**2.16 (1.30–3.59)**	14 (5.7%)	
51–64 yrs	189	76 (40.2%)	**3.17 (1.86–5.39)**	10 (5.3%)	
≥65 yrs	43	14 (32.6%)	**2.27 (1.03–4.99)**	2 (4.7%)	
Sex					
Female	781	239 (30.6%)	1.0	56 (7.2%)	N.S. and NIM
Male	382	159 (41.6%)	**1.62 (1.24–2.10)**	18 (4.7%)	
BCG status					
Not vaccinated	684	203 (29.7%)	1.0	44 (6.4%)	N.S. and NIM
Vaccinated	479	195 (40.7%)	** 1. 79 (1.38–2.34)**	30 (6.3%)	

TST: tuberculin skin test; QFT-G: QuantiFERON-TB Gold test; *n*: number in subset of *N*; aOR: adjusted odd ratio; 95% CI: 95% confidence interval; N.S.: not significant; NIM: not in model; BCG: bacille Calmitte-Guérin; QFT: QuantiFERON-TB test.

*Multivariate models were created using factors with *P* values ≤0.2 in univariate analysis and <0.05 in stepwise logistic regression until the best fitting, parsimonious model was identified. Model fit was evaluated using the Hosmer-Lemeshow test. **Bold font** indicates statistically significant aORs.

**Data from 83 subjects with indeterminate QFT-G results were excluded from analysis.

**Table 6:** Subject characteristics associated with CXR and TST discordance.

Characteristic	*N*	CXR positive but TST negative		CXR negative but TST positive	
		*n*	aOR* (95% CI)	*n*	aOR* (95% CI)
Total	1163**	79		501	
Age group in years					
18–20 yrs	130	6 (4.6%)	1.0	42 (32.3%)	1.0
21–30 yrs	270	3 (1.1%)	**0.23 (0.06–0.94)**	120 (44.4%)	**1.90 (1.22–2.98)**
31–40 yrs	279	7 (2.5%)	0.53 (0.17–1.59)	161 (57.7%)	**2.91 (1.87–4.53)**
41–50 yrs	252	19 (7.5%)	1.59 (0.62–4.11)	103 (40.9%)	1.54 (0.98–2.42)
51–64 yrs	189	32 (16.9%)	**3.88 (1.57–9.62)**	69 (36.5%)	1.38 (0.85–2.25)
≥65 yrs	43	12 (27.9%)	**6.76 (2.31–19.79)**	6 (14.0%)	0.39 (0.15–1.004)
Sex					
Female	781	51 (6.5%)		324 (41.5%)	1.0
Male	382	28 (7.3%)	N.S. and NIM	177 (46.3%)	**1.33 (1.03–1.73)**
Other major medical conditions					
No	1146	72 (6.3%)	1.0	497 (43.4%)	
Yes	17	7 (41.2%)	**4.52 (1.58–12.96)**	4 (23.5%)	N.S. and NIM
BCG status					
Not vaccinated	684	58 (8.5%)		253 (37.0%)	1.0
Vaccinated	479	21 (4.4%)	N.S. and NIM	248 (51.8%)	**1.61 (1.25–2.07)**
*M. avium* reactivity					
No	812	58 (7.1%)		369 (45.4%)	1.0
Yes	351	21 (6.0%)	N.S. and NIM	132 (37.5%)	**0.71 (0.54–0.93)**

CXR: chest radiograph; TST: tuberculin skin test; *n*: number in subset of *N*; aOR: adjusted odd ratio; 95% CI: 95% confidence interval; N.S.: not significant; NIM: not in model; BCG: bacille Calmitte-Guérin; QFT: QuantiFERON-TB test.

*Multivariate models were created using factors with *P* values ≤0.2 in univariate analysis and <0.05 in stepwise logistic regression until the best fitting, parsimonious model was identified. Model fit was evaluated using the Hosmer-Lemeshow test. **Bold font** indicates statistically significant adjusted odds ratios (aORs).

**Data from 83 subjects with indeterminate QFT-G results were excluded from analysis.

**Table 7:** Subject characteristics associated with CXR and QFT-G discordance.

Characteristic	*N*	CXR positive but QFT-G negative		CXR negative but QFT-G positive	
		*n*	aOR* (95% CI)	*n*	aOR* (95% CI)
Total	1163**	164		262	
Age group in years					
18–20 yrs	130	8 (6.2%)	1.0	24 (18.5%)	1.0
21–30 yrs	270	7 (2.6%)	0.36 (0.12–1.02)	69 (25.6%)	1.52 (0.90–2.55)
31–40 yrs	279	18 (6.5%)	1.02 (0.43–2.43)	72 (25.8%)	1.54 (0.92–2.58)
41–50 yrs	252	47 (18.7%)	**3.16 (1.44–6.95)**	60 (23.8%)	1.38 (0.82–2.34)
51–64 yrs	189	64 (33.9%)	**7.60 (3.49–16.53)**	35 (18.5%)	1.004 (0.565–1.784)
≥65 yrs	43	20 (14.1%)	**13.26 (5.22–33.72)**	2 (4.7%)	**0.215 (0.05–0.95)**
Prior TB					
No	1157	160 (13.8%)	1.0	262 (22.7%)	
Yes	6	4 (66.7%)	**16.26 (2.42–109.33)**	0 (0.0%)	N.S. and NIM
Chest exam					
Normal	1157	160 (13.8%)	1.0	262 (22.7%)	
Abnormal	6	4 (66.7%)	**20.81 (2.76–156.68)**	0 (0.0%)	N.S. and NIM

CXR: chest radiograph; QFT-G: QuantiFERON-TB Gold; *n*: number in subset of *N*; aOR: adjusted Odd Ratio; 95% CI: 95% confidence interval; TB: tuberculosis; N.S.: Not Significant; NIM: Not in model.

*Multivariate models were created using factors with *P*-values ≤0.2 in univariate analysis and <0.05 in stepwise logistic regression until the best fitting, parsimonious model was identified. Model fit was evaluated using the Hosmer-Lemeshow test. **Bold font** indicates statistically significant adjusted odds ratios (aORs).

**Data from 83 subjects with indeterminate QFT-G results were excluded from analysis.
